# Genito-Urinary Surgery

**Published:** 1905-02-18

**Authors:** 


### PROGRESS IN MEDICINE AND SURGERY.

### GENITO-URINARY SURGERY.

**Lithotrity for Large Vesical Calculi.**—Frank Milton[1] has discussed the choice of operation for large vesical calculi. In the case of small stones the choice of operation is not of great moment, for any operation should be successful. The author considers lithotrity offers distinct advantages for large calculi of 50 grammes weight and over (or 750 grs. and over). Statistics of the operations performed by H. M. N. Milton (113 cases) and Frank Milton (46 cases), show a mortality of 5·9 per cent. for urethral lithotrity, of 14·9 per cent. for perineal lithotrity, and of 36·3 per cent. for suprapubic lithotomy. Six cases were treated by lateral lithotomy without a death. The statistics show a mortality in favour of crushing without a wound of 13 per cent. The cases recorded were dealt with in Egypt. The instruments used were those devised by H. M. N. Milton. By perineal lithotrity is meant the operation of Reginald Harrison, in which the prostate is incised and the stone broken up sufficiently to allow of its removal by forceps, not the operation of Keith, in which a small incision is made into the membranous urethra and the stone crushed by a larger lithotrite than can be passed down the unopened urethra.

**Repair of the Urethra by Transplantation of the Urethra of Animals.**—J. Hogarth Pringle[2] describes three cases in which, to remedy defects in the urethra, he grafted into the tissues portions of the urethra of the ox. Two of the patients were men who had had extensive rupture of the perineal part of the urethra. The third was a boy with hypospadias and deficiency of the floor of the penile portion of the urethra. In rupture of the urethra, unless the rupture is repaired by the union of urethral epithelium, intractable stricture is sure to follow. Guyon is quoted as advocating the treatment of these cases by freeing the two ends of the urethra and allowing them to heal, first over a catheter, tied in for a few days, and then without a catheter. The soft tissues of the perineum are sutured over the urethra. It is claimed that a regeneration of the mucous membrane of the urethra is attained in this way. Another plan is to employ a skin or mucous membrane flap or graft, and to convert this into a canal by a second operation. For the method now particularly described by the author the penis of a young bullock, just killed, is obtained and conveyed to the operating-room in hot sterile salt solution. From this the urethra is dissected out and a sufficient length of it transplanted and sutured to the ends of the urethra, previously freed, bounding the deficiency. The method was employed five times in three cases. The grafted tissue lived each time and retained a patent channel. In two patients the junction of the graft to the penile end of the urethra did not completely heal. In the hypospadias case the result, after a second grafting operation, and after closing a fistula at the place where the graft joined the glans penis, was excellent.

The Treatment of Ruptured Urethra.—H. Rutherford [3] discusses this subject and reports a series of seven successful cases. The method of treatment this writer advocates is by incision down to the injured urethra to evacuate clots and inflammatory products, and by combined drainage of the bladder with a suprapubic tube and a soft catheter in the urethra. In one case some leakage of urine occurred by the side of the catheter and the perineal wound did not heal as in the others and a stricture formed. The stricture was resected and the ends of the urethra sutured and the bladder drained suprapubically. Seven years later the urethra admitted a No. 13–16 bougie without difficulty. The writer believes that suturing the urethra is unnecessary with the procedures mentioned above, when the divided ends of the urethra lie in contact on a soft catheter with the limbs extended, though they may be widely separated with the patient in the lithotomy position. There appears to be no great tendency of the two ends to retract. The catheter used should be a soft one and the largest the urethra will admit and is a valuable instrument for bringing the divided ends into proper contact and keeping open the lumen of the urethra.

The Treatment of Involuntary Micturition (incontinence).—Professor R. Kutner (Berlin) [4] has discussed the causation and treatment of involuntary and unconscious micturition. The term involuntary micturition may be used in two senses, viz, to mean micturition which cannot be avoided in spite of conscious efforts, or micturition accomplished unconsciously. It is a particular form of the latter variety of involuntary micturition, dependent upon local muscular anomalies which the author specially describes. As an example the case is cited of a girl, seven years old, who ever since birth had unconsciously wet her clothes with urine every two or three hours by day and by night. The effects were grievous both for the patient and for those who came near her. Repeated catheterisations established the facts that the bladder always contained too much urine and that the sphincter vesicæ was in a state of chronic spasm which strongly opposed the entrance of the catheter into the bladder. The troubles were considered to have arisen in the first instance from over-distension of the bladder and weakening of the detrusor muscle. The normal antagonism between the detrusor and the sphincter muscles was thus disturbed and the latter gained the upper hand. A vicious circle was established with further weakening of the detrusor and strengthening of the sphincter. The result was a form of spasmodic overflow incontinence and the retention of residual urine in the bladder. The writer believes the above explanation applies to nearly all cases of enuresis nocturna et diurna in children and to the majority of cases of unconscious and involuntary micturition in adults. Bed-wetting in isolated cases, it is admitted, may occur without distension of the bladder, depending on psychical causes and must be then treated by psychical methods, such as hypnotism, as well as by the methods given below. Women, with the form of incontinence under consideration, often complain that when walking they suddenly find that they have wet their clothing, or they have done this in the sitting posture when sneezing, coughing, or laughing. In men incontinence of this kind is rare. In children and adults the cause is the same—namely habitual or frequent undue postponement of the act of micturition, leading to weakening of the detrusor and strengthening of the sphincter. The treatment has for its object the weakening of the sphincter and the strengthening of the detrusor, and consists in the gradual dilatation of the neck of the bladder with metal sounds and in making the patients micturate at regular intervals, every two or three hours, whether there is a desire to do so or not. Such treatment, it is said, will in nearly all cases effect a cure of this form of unconscious and involuntary micturition.

Acquired Phimosis.—Professor Rille (Leipzig) [5] has written on this subject. Symptomatic phimosis is usually the result of inflammatory processes. It has been alleged by some that congenital phimosis is the result of laceration of the preputial margin soon after birth. Congenital phimosis is a disposing cause for balanitis, papillomata, and erosions. The assertion that phimosis specially favours the acquisition of venereal disease is denied by the author; on the contrary, phimosis and the collection of smegma it favours give protection against infection from vaginal secretions. The most frequent causes of acquired phimosis are balanitis, gonorrhœa, and chancres. To exclude gonorrhœa in the diagnosis, the preputial cavity must be thoroughly cleansed with a syringe armed with a thin rubber tube, and the urine then collected and examined for evidence of gonorrhœa. In one form of balanitis, probably a contagious form, a special spirillum has been found, together with other organisms. In balanitis the prepuce is reddened at the edge and at the level of the corona glandis; in gonorrhœa a soft œdema extending far into the penis is often found. In some gonorrhœa cases a red band-like streak, with here and there nodules in it, is present in the dorsum penis. This feels like a swollen lymphatic duct. It is less tender, less defined, and softer than the indolent and sclerosed lymphatic felt in a similar position in syphilis. Tenderness of the glands in the groin is associated with the lymphangitis of gonorrhœa. Similar lymphangitis may be met with in balanitis if ulceration and erosion is present. The phimosis from soft chancres is associated with more intense inflammation. With the phimosis of primary syphilis there is frequently indurative œdema, and the penis appears elongated as well as thickened. Phimosis from diabetic balanitis is so characteristic, that a diagnosis of diabetes may be made from it before the urine has been examined. The prepuce is thickened and hardened, and this prevents its retraction, though the terminal opening may seem wide enough. The margin is fissured, or shows cicatrices. Small roundish ulcers are present. The mycelium of a fungoid growth is found in the retained secretion. In these cases other diabetic symptoms are often absent, and the quantity of sugar in the urine is often small. The fear of gangrene following circumcision in diabetic phimosis is not justified. Circumcision for most forms of inflammatory phimosis is not necessary. Syringing beneath the prepuce and fomentations will often succeed in reducing the phimosis in the absence of a congenital narrowing. Gauze strips may be used for draining the cavity, and the preputial orifice may be dilated by compressed sponge tents, of the thickness of match sticks, wrapped round with gauze. With regard to paraphimosis, the writer points out that in the symptomatic form there is no gangrenous ulceration in the constricting band. In the traumatic form after three or four days, a muco-purulent urethral discharge occurs which may be confused with gonorrhœa. It is probably produced by circumscribed inflammation at the seat of constriction.

[1] Lancet, Oct. 1, 1904, p. 949. [2] Annals of Surg., Sept. 1904, p 387. [3] Lancet, Sept. 10. 1904, p. 751. [4] Zeitschrift f. ärztliche Fortbildung, Nov. 15, 1904, p. 650. [5] Deutsche Medizinische Wochenschrift, Nov. 24, 1904, p. 1767.

(*To be continued.*)

